# The WHO Algorithm for Causality Assessment of Adverse Effects Following Immunization with Genetic-Based Anti-COVID-19 Vaccines: Pitfalls and Suggestions for Improvement

**DOI:** 10.3390/jcm13237291

**Published:** 2024-11-30

**Authors:** Paolo Bellavite, Alberto Donzelli, Ciro Isidoro

**Affiliations:** 1 Independent Researcher, 37134 Verona, Italy; 2 Foundation Allineare Sanità e Salute, via Ricordi 4, 20131 Milano, Italy; info@fondazioneallinearesanitaesalute.org; 3 Department of Health Sciences, Università del Piemonte Orientale, Via P. Solaroli 17, 28100 Novara, Italy

**Keywords:** COVID-19 vaccine, vaccine safety, adverse effects following immunization, pharmacovigilance, causality assessment, WHO algorithm, differential diagnosis

## Abstract

Clarifying and differentiating the causes of diseases is an essential step in any clinical activity, but it takes on particular relevance and complexity in the case that arise following vaccinations. The WHO has proposed a protocol that uses a list of specific questions about vaccine-related adverse events and an algorithm for making a judgement. Here, we analyze and discuss the important limitations of this protocol when applied to the new genetic-based anti-COVID-19 vaccines, particularly once dealing with rare and unexpected pathological events. The main controversial aspects concern: (a) the prevailing consideration of other possible causes; (b) the biological plausibility and the choice of an appropriate time window to consider adverse effects possibly caused by vaccines; (c) the reference to scientific literature, which may be very limited and often controversial in early stages of introducing new vaccines because of the short period of observation; (d) the final classification of the algorithm into only three classes, which leaves ample space for the “indeterminate” category. Failure to address these issues may lead to distorted pharmacovigilance reports with significant consequences on the benefit/harm assessment. In anticipation of possible future pandemics managed with new vaccines, the WHO algorithm needs to be revised with appropriate protocols for monitoring and evaluation of adverse effects that take into account the novel mechanism of action and real-world epidemiological data.

## 1. Introduction

To tackle the COVID-19 pandemic, and in the hope to stop the virus spreading, in 2021, governments across the world implemented mass vaccination with a novel class of genetic pro-immunogenic products, based on modified mRNA [1,2,3] or adenoviral vectors [4,5]. After only a few months of clinical trials with a relatively small number (~43,000 and 30,000) of healthy volunteers, those vaccines were given emergency approval, and were then rapidly deployed to the whole population, including vulnerable patients with co-morbidities, pregnant women, children, and even naturally post-infection immunized individuals [6]. The anti-COVID-19 genetic pro-vaccines were found effective in attenuating the severity of the disease [2,7,8], though the maintenance of such protection requires a periodic boosting [9,10]. Yet unfortunately and contrary to the early assumption, these genetic pro-vaccines were less effective in stopping transmission of SARS-CoV2, even between fully vaccinated contacts [11,12,13].

Vaccines are generally considered safe enough to be given to healthy individuals, even at a very young age. However, some of the vaccinees may experience adverse reactions sooner or later [14,15]. Besides the clinical trials needed for their registration, the safety of vaccines is investigated through many different approaches, of which very important ones are the post-marketing pharmacovigilance of the incidences of adverse events following immunization (AEFI) and the assessment of causality, problems which are closely linked to each other. This could be tricky, because the adverse event may be very rare or undetected in healthy volunteers in the vaccinated arm of the clinical trial. They may emerge once the vaccine is administered to a large population, also including patients with a variety of different morbidities, as was the case with the COVID-19 vaccines. Determining the possible role of the vaccine in causing AEFI is essential to provide the population with reliable benefit/risk assessments and to maintain the confidence in vaccines and health agencies. Moreover, this study has important implications in the compensation programs for serious and permanent injuries rarely caused by vaccines, provided for by national legislations [16].

An AEFI is defined as “any untoward medical occurrence which follows immunization and which does not necessarily have a causal relationship with the use of the vaccine” [17]. It is therefore essential to investigate the possible correlation between vaccine and adverse event, both at individual and at population levels. In the first case, the question is “Did the vaccine given to a particular individual cause the particular event reported?”. Answering this question is important for both the person involved and for clinical (e.g., risk of further reactions) and medico-legal (e.g., government compensation programs) reasons. As highlighted in the WHO pamphlet [17], it is rarely possible to get a direct answer to this question in a single case, so in most cases the assessment ends with a probability that the two events are correlated or not. At the population level, the question is “Can the given vaccine cause a particular adverse event?” (i.e., “Can it?”). Population-level assessments and vaccine monitoring are conducted during vaccine deployment through various systems such as the Vaccine Adverse Event Reporting System (VAERS) [18], V-safe, and the Vaccine Safety Datalink (VSD) in USA (https://www.cdc.gov/vaccine-safety-systems/about/cdc-monitoring-program.html, accessed on 7 November 2024), the Yellow card in UK (https://www.gov.uk/guidance/the-yellow-card-scheme-guidance-for-healthcare-professionals, accessed on 7 November 2024), SmartVax in Australia, and the Canadian National Vaccine Safety (CANVAS) Network in Canada [19]. Epidemiological methods for safety assessment are also applied for specific research designs, such as cohort, case-control, and self-controlled case series (SCCS) [20]. Finally, an outstanding relevance of the “real world” safety assessment assumes post-marketing pharmacovigilance, which in turn may be passive (spontaneous) or active (usually based on questionnaires or similar tools, and reminders for the non-respondents) [21,22,23,24,25]. In any case, proper registration of pharmacovigilance data needs a causality assessment in each case report, particularly those concerning adverse events of special interest and deadly events.

In the particular case of COVID-19, the real incidence of adverse events correlated to vaccines is difficult to determine for two main reasons: (a) the vaccine surveillance system in most countries (including Italy) is typically passive and it has been proven largely inefficient, as discussed elsewhere [26,27], and (b) when dealing with a new-generation vaccine, safety signals can only be collected after it has been widely distributed among different populations and when there is sufficient knowledge of the pathogenetic mechanisms that can explain the causal link with the adverse events [28]. An example of a causality assessment framework has been formulated by the Korean National Academy of Medicine, which is based on epidemiological and mechanistic evidence [29]. However, it has been pointed out that the lack of a clear biological mechanism (which is unique for each type of vaccine) should not be taken as a pretext to exclude a priori a causal association particularly when knowledge on the vaccine’s pharmacodynamics and pharmacokinetics is still limited [30]. Thus, the experimental and epidemiological studies aimed at determining or excluding a causal link must clearly specify the criteria and determinants used [30]. Causality assessment is a complex and difficult procedure, requiring multiple interdisciplinary skills [17], which gives rise to additional elements of uncertainty. This last aspect concerns, in particular, post-vaccination serious adverse events, such as deaths. For example, in the evaluation of deaths after the anti-COVID vaccine reported in the first two years (2021–2022) to AIFA, the Italian medicine agency data submissions labeled as much as 28.0% “indeterminate” and 9.1% “unclassifiable” (see Section 4 below). Here, we focus on these crucial aspects and review the recent literature that has addressed these issues.

## 2. Inconsistency in Pharmacovigilance

To monitor the safety of a novel vaccine largely deployed in an infectious emergence scenario, a proactive and reinforced pharmacovigilance system should be mandatory [31,32,33,34]. In this respect, efforts were made in many countries to proactively enhance passive surveillance for AEFI and to initiate studies to collect data to evaluate the possible causality of adverse events [29,35]. Unfortunately, this was not put in place in all countries in due time. Randomized clinical trials (RCT) can only identify the most common adverse effects, especially if they are not designed to identify specific events and if the observation period is limited to a few months, which is insufficient for the emergence of chronic effects. However, running RCTs to assess the safety of a vaccine during the pandemic was not feasible (or could be considered unethical) [30]. Therefore, further safety evidence should come from population-based phase IV studies and pharmacovigilance systems. The need for post-marketing adverse reaction surveillance systems for new COVID-19 vaccines has been recognized by health authorities, but the platforms used by different countries were different and inadequate to ensure the safety assessment of these vaccines [36].

Many new and unexpected adverse reactions to COVID-19 vaccines appeared in the first few months of rollout. The most frequent of them were thrombosis (especially venous) [37], myocarditis/pericarditis [38], vasculitis and inflammatory dermatological reactions [39], and disorders of the menstrual cycle [40]. The latter is a perfect and particularly significant case study for the topic we are dealing with. Indeed, any causal link between menstrual disorders and vaccination has long been denied due to the high prevalence of the phenomenon in the population, which has delayed its recognition as an alarm signal by pharmacovigilance systems. Epidemiological studies have subsequently confirmed the signal, so much so that the EMA recommended its inclusion among the adverse effects of vaccines in the product information of Comirnaty and Spikevax on 27 October 2022, almost two years after the start of the vaccination campaign (https://www.ema.europa.eu/en/news/meeting-highlights-pharmacovigilance-risk-assessment-committee-prac-24-27-october-2022?utm_medium=email&utm_source=substack, accessed on 20 September 2024). Many other diseases and organ disorders are reported in the pharmacovigilance databases, as well as a higher number of serious adverse events than those reported for other vaccines [41]. The correlation between several unusual adverse events and vaccines is still under investigation.

An example that clearly illustrates the unreliability of the passive pharmacovigilance system concerns the data released by AIFA, the Italian medicine agency, which, in its 12th report, states that the serious adverse effects were 18.1 per 100,000 doses, of which only one third could be considered vaccine-related [42]. This strikingly contrasts with the results of phase III RCTs conducted by the pharmaceutical companies, where the serious adverse events associated with the two mRNA vaccines reported in the vaccine group were 980 per 100,000 participants [14], about 54 times higher than the total number declared by AIFA passive pharmacovigilance. Note that compared with placebo, the two mRNA vaccines taken together were associated with an excess of serious adverse events of special interest: Risk Ratio 1.43 (95% CI 1.07 to 1.92) [14]. In some studies, the difference of reported severe adverse reactions and events between passive and active pharmacovigilance was about 1 to 1000.

A fairly accurate estimate of the difference between passive and active vigilance is obtained by comparing data from the VAERS system (spontaneous reporting) with the V-safe system (active monitoring) [43]. In the VAERS system, reports of serious adverse events in adolescents aged 12 to 17 years after receiving the Pfizer-BioNTech vaccine were 1726 out of 32,268,525 doses administered (reporting rate = 5.3/100,000). The V-safe system reported data from 172,032 adolescents aged 12 to 17 who completed symptom monitoring within a week of the injection. Among these, approximately 4% were unable to attend school, 3.1% complained of severe fatigue, and 0.6% needed medical treatment. The latter figure (0.6%) represents a rate of 600/100,000, more than 100 times higher than the rate of serious adverse events reported through VAERS.

Examples of active pharmacovigilance for vaccine-related adverse effects are the blinded phase of mRNA-1273 SARS-CoV-2 vaccine trials in adults [2] and in adolescents [44]; V-safe [[45]—Table 5]; active surveillance for myocarditis and pericarditis [46,47,48,49] and for measles-mumps-rubella-chickenpox vaccine [32,50].

The above example leads to the assumption that evaluation of benefit/risk balance of vaccines should not be based on the passive pharmacovigilance data only, neglecting their intrinsic methodological limits. In fact, passive pharmacovigilance is useful for the discovery of “safety signals” that emerge from the widespread use of vaccines. In fact, a safety signal is “an information which suggests a new and potentially causal association, or a new aspect of a known association, between an intervention and an event or set of related events, either adverse or beneficial, that is judged to be of sufficient likelihood to justify verificatory action” [17], but it cannot have a quantitative value on an epidemiological level. As noted above, several pre-clinical and clinical studies, post-licensure analytic studies, as well as post-licensure pharmacovigilance concur in the overall evaluation of vaccine safety. It should also be noted that the long-term or chronic effects (positive or negative) of vaccines on the population as a whole can be evaluated by epidemiological studies such as the all-cause or cardiac-related mortality in self-controlled case series and matched case-control studies [51,52,53,54]. As discussed above, the assessment of possible causal correlation is always performed on individual cases of AEFI, which in most cases cannot lead to a certain identification of the determining cause (vaccine or an independent or another random factor) due to the complexity of the clinical situation. This, however, does not justify “excluding” the causality of a series of single cases.

In short, if the safety assessment of vaccines were based on passive pharmacovigilance and the exclusion of correlation (due to uncertainty), it would follow that any conclusions on the benefit/harm balance would be unreliable, due to underestimation of the denominator.

## 3. WHO Criteria for Causality Assessment

Multiple criteria and algorithms are available for establishing a causal relationship between a therapeutic drug and an adverse drug reaction (ADR) that essentially consider the following clinical-pharmacological aspects of the case history: (i) dose and time correlation between drug administration and the appearance of the event; (ii) biological plausibility based on the pathological mechanisms; (iii) possible alternative causes; (iv) consistency with previous reports in the literature; and (v) dechallenge/rechallenge effects (i.e., withdrawal and reintroduction of the drug), when possible [32,55,56,57]. As we will discuss below, not all these criteria can be applied to the vaccines, such as the dose-dependency or the dechallenge/rechallenge criterion. Several methods exist to assess the causality of AEFIs, which do not always agree with each other [58,59,60,61]. The method that eventually proved to be prevalent is the one developed by the WHO in the last decade [55,62,63,64,65]. The method progressively analyzes the various aspects of the issue, eventually allowing reported events to be divided into three groups: consistent causal association, inconsistent causal association, and indeterminate [17].

**Figure 1:**
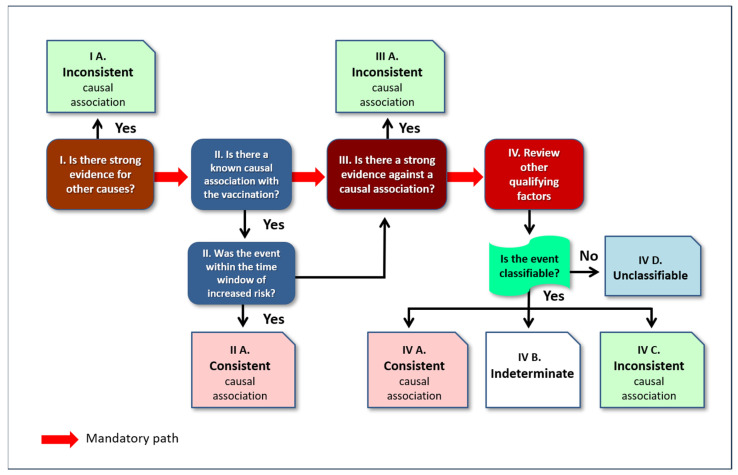
Causality assessment algorithm according to WHO manual [17]. Re-drawn under the Creative Commons Attribution-Non Commercial-Share Alike 3.0 IGO license.

The entire procedure is summarized in the algorithm represented in Figure 1.

The conceptual premise of causality analysis is well expressed at the beginning of the cited manual: “Causality is the relationship between two events (the cause and the effect), where the second event is a consequence of the first. A direct cause is a factor in absence of which the effect would not occur (necessary cause). Sometimes there are multiple factors that may precipitate the effect (event) or may function as co-factors so that the effect (event) occurs.” These important conceptual expressions seem simple and straightforward, but they are not easy to apply in the field of vaccinology for the following reasons.

The definition of a “direct cause” implies that the vaccine must be the only direct and “necessary” factor in causing the adverse event, which is difficult or even impossible to prove. In other words, only those diseases that specifically manifest themselves solely because of vaccination would have to be considered. But what might these diseases be? Certainly not the most observed ailments such as hyperpyrexia, muscle fatigue, arthralgia, headache, thrombosis, autoimmunity, myocarditis, and others more serious, up to sudden death. These clinical conditions can also be caused by other factors and occur even in the absence of the vaccine. There are objectively few and rare diseases that would not occur in the absence of vaccination. Therefore, by this criterion, the vaccine can never be considered the “direct” and sole cause of the common ailments complained of by vaccinees.

In fact, the above criteria apply to a disease caused only by a vaccine, an absolutely unlikely condition! The other condition is a test proving not only the presence of the vaccine material (which is obvious, if it was injected into a body), but also that the specific material was the cause of the event. In fact, the cited manual states that “For this reason, a defined causal association or absence of association often cannot be proven or disproved for an individual event”.

It follows that virtually all adverse reactions to vaccines fall into the second category, in which the vaccine is only one of the triggers for the event, in the presence of individual susceptibilities, due to other more or less identifiable factors. For these reasons, it is essential to establish the extent of the role of the vaccine as a co-factor and the possibility of an interactive causality of multiple events.

In the next paragraph, we review the main points of the WHO protocol and discuss whether and how they apply to COVID-19 vaccines.

## 4. The First and Crucial Step: Is There Another Cause?

**Figure 2:**
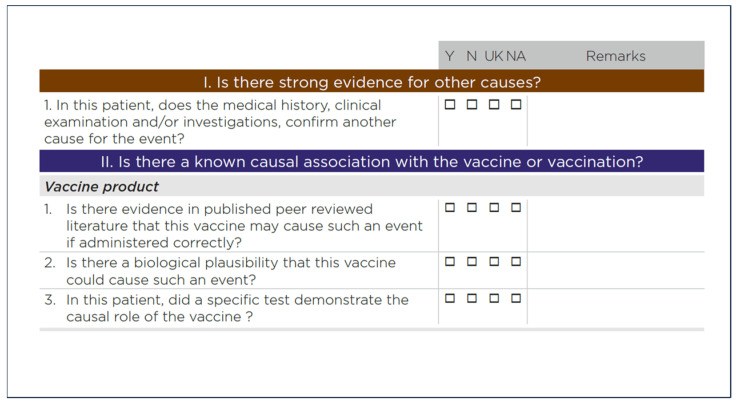
First two steps of the WHO protocol for causality assessment and part of the related “checklist” [17]. This work is available under the Creative Commons Attribution-NonCommercial-ShareAlike 3.0 IGO license.

The WHO manual recommends answering a checklist, as shown in Figure 2.

The first question, “*Is there strong evidence for other causes?*”, implies that if there are other “strong” causes, the vaccine is exonerated. This is a seemingly logical question, but poorly posed, because it does not consider interactions between multiple causes, which instead might be the case [56,57].

The WHO scheme specifies that a causal association between vaccine inoculation and an AEFI is “*A cause-and-effect relationship between a causative factor and a disease with no other factors intervening in the process*”. Though formally correct, this definition excludes the possibility that the causal association arises from multiple and interacting factors, including the vaccine.

As an example, let us take a close look at how the WHO method has been applied by the Italian agency, AIFA, with regards to the data on the deaths. In the period 26 December 2020 (the start of the vaccination campaign) through 26 December 2022, AIFA registered 971 AEFI with death as the outcome [66], a number possibly underestimated because it is derived from spontaneous reports. By applying the WHO algorithm [17], AIFA concluded that: “*59.4% of cases are not correlated, 28.0% are indeterminate and 9.1% are unclassifiable due to lack of sufficient information. Overall, 29 cases out of the 812 (3.6%) evaluated were linked to the anti-COVID-19 vaccination*” [66] and that “*the evaluations of the cases suggest the absence of responsibility of the vaccine in the majority of these, as they are often subjects with intercurrent or previous pathologies, with clinical frailties, such as: cardiovascular, metabolic, oncological, autoimmune, neurodegenerative, respiratory, renal, hepatic, pancreatic, lymphopoietic system (coagulation defects)*” [67].

Remarkably, the diseases listed include those most commonly represented in the Italian population. Thus, by applying the WHO protocol, those who died after the vaccine and were carriers of “other diseases” did not die from the vaccine, but from the diseases they had before. This is a very serious conceptual error, because it ignores any possibility of the vaccine being a trigger in people “with clinical vulnerability”. In other words, the possibility that those patients could live longer if not vaccinated for COVID-19 is not considered. Unfortunately, a cohort of (unvaccinated) controls has never been considered for comparison. Also, it is to be stressed that those “vulnerable” patients were strongly recommended to get vaccinated with priority, and to be vaccinated several times.

Most importantly, the misapplication of the algorithm in these patients emerges when considering that many of the diseases considered to be the real cause of death (e.g., cardiovascular, autoimmune, and lymphopoietic system diseases and coagulation defects) could instead be a consequence of the COVID-19 vaccination [68,69,70,71,72,73]. Additionally, an analysis of mRNA COVID-19 vaccines reported to VAERS showed that patients with underlying cardiovascular disease conditions were significantly more likely to experience serious cardiovascular adverse events than people without such conditions [74]. Thus, despite the many studies indicating that vaccination confers some protection from deadly events in patient with co-morbidities, it is still necessary that the causality assessment is carried out correctly in each individual case, without discarding the accountability of the vaccine in cases where there is a pre-existing risk factor, which could be confused with the hypothetical effect of the vaccine itself. If applied systematically to individual cases, such misunderstanding could lead, in a large population, to an underestimation of the vaccination risk for large categories of people affected by pre-existing diseases.

When dealing with multifactorial diseases, a susceptible condition should not be taken tout-court as the cause of the fatal event, and the possibility that intervening factors act as triggers that precipitate the clinical condition should be considered. This is particularly relevant when the intervening factor can directly or indirectly affect the diseased organ (as could be the case for COVID-19 vaccines).

In this context, an emblematic example is offered by the WHO manual (Annex 2, page 61), where it presents a case of meningoencephalitis with convulsions that began five days after immunization with the anti-meningococcal conjugate vaccine. Since the analysis of the cerebrospinal fluid revealed the presence of herpes simplex virus, the association of meningoencephalitis with the immunization was judged as “inconsistent” following the first step of the algorithm. However, this conclusion ignores the possibility of reactivation of herpes virus following vaccination, an eventuality already encountered several times in the case of COVID-19 genetic vaccines [75,76,77,78,79], and previously for hepatitis A, rabies, and trivalent influenza vaccines [80].

Thus, one should consider whether that *particular* vaccine and the vaccination schedule possibly cause a transient immunosuppression state that precipitated a latent infection or other immune-dependent disease.

## 5. What Is Known About the Vaccine Product

The second step of the causality assessment involves asking several questions about the vaccine product. Of particular interest are the first three questions (Figure 2), which deserve further comments.

### 5.1. Is There Evidence in Published Peer Reviewed Literature That This Vaccine May Cause Such an Event if Administered Correctly?

Obviously, this question makes sense only after the vaccines have been used for some time, and certainly not at the experimental stage and in the early period of roll out. More precisely, it would make sense if the answer was positive, that is, if the risk of this adverse event has already been reported in the literature. On the other hand, a negative answer (lack of evidence) cannot be considered as “evidence of absence”, because rare adverse events related to vaccination could occur after a long period of vaccine use in many people. Thus, this question should be rephrased or include cautions in the “checklist” about the meaning of a negative response when applied to newly introduced vaccines.

Regarding anti-COVID-19 vaccines, a picture of the adverse effects caused by vaccination has only emerged in the literature with time. The risk of vaccine-associated immune thrombosis and thrombocytopenia (VITT) was recognized as rare adverse effects of COVID-19 adenoviral vaccines in the first weeks of April 2021, i.e., over three months after the roll-out of vaccines [81,82]; myocarditis and pericarditis complicating mRNA vaccines, especially in young males, were recognized in June 2021 [83]; a potential small but statistically significant safety concern for Guillain-Barré syndrome was published in October 2021 [84]; menstrual irregularities after COVID-19 vaccination were denied during 2022 [85] but were recognized in 2023 [86]. These examples, among the many possible ones, suggest that the lack of evidence in the literature cannot be relied on to exclude a possible correlation between an adverse event and inoculation with a new vaccine. A clarification in the checklist associated with the WHO algorithm would also be useful in this particular aspect.

### 5.2. Is There a Biological Plausibility That This Vaccine Could Cause Such an Event?

In pharmacology, the biological plausibility of a causal link, that is, how credible, convincing, and/or logical the cause-and-effect relationship is, depends on the knowledge of the mechanism of action of the product used and its pharmacokinetics and pharmacodynamics. Again, it appears reasonable that the biological plausibility of adverse reactions caused by a new vaccine may emerge over time and as studies deepen. For example, many of the biological effects of the spike protein were not even known for that of the virus and were not studied further until after the vaccines were distributed [87]. Initially, it was believed, or at least stated, that the lipid nanoparticles would remain “in situ” [88]. Therefore, any adverse events could only be attributed to local or systemic reactions caused by the immune response, such as fever, fatigue, or arthralgia, as is the case with other vaccines. Later, it was discovered that the vaccine (genetic) material could affect all organs, including the heart. This discovery made cardiovascular reactions such as hypertension, myocarditis, pericarditis, and autoimmune diseases more plausible [72,89,90]. Coagulation disorders, such as thrombosis, have become plausible due to the discovery of antibody reactions against the spike protein and the soluble ACE2 complex [91,92] or the discovery of anti-PF4 or other antibodies in the plasma of vaccinated persons [93,94,95].

The issue of plausibility is also relevant for pharmacovigilance reporting. For vaccines developed with new technologies, it is conceivable that unexpected adverse events will eventually appear that were not described for conventional vaccines. For instance, at the beginning of the vaccination campaign, many reports of thrombotic phenomena were considered to be random or unrelated, because it seemed impossible (implausible) that vaccines could cause thrombosis. Yet, later, the European agency EMA confirmed a possible causal link between AstraZeneca’s vaccine and very rare cases of unusual blood clots with low blood platelets. Thus, it would be wise to actively report all AEFIs with a new vaccine, not just those considered plausible based on previous experience with other vaccines.

### 5.3. The Time Window

Plausibility also relates to the ‘time window’ mentioned in step II of the WHO protocol. In the case of a new vaccine, one cannot establish a time frame within which the adverse reactions can be considered until the observation of various danger signals has been consolidated. For instance, autoimmune reactions usually show up long after (weeks to years) the initial exposure to the triggering substance. Autoimmune diseases are dependent on various predisposing factors, including genetics, gender, other diseases, drugs, and previous or subsequent SARS-CoV-2 virus infection.

The causal relationship between COVID-19 vaccination and autoimmunity is controversial and difficult to assess because the autoimmune consequences may become clinically apparent years after vaccination. Moreover, COVID-19 vaccinations can be followed by subacute or chronic pathological conditions, possibly with an autoimmune background [72,96,97,98], which makes the causality assessment in individual cases more challenging, particularly when the clinical symptoms do not manifest soon after the vaccination but after a long period and in conjunction with confounding co-factors. On the one hand, COVID-19 vaccination was found to reduce the risk of developing various autoimmune diseases brought about by SARS-CoV2 infection [99]. In addition, a large Korean population-based cohort study conducted for one year concluded that the risk for most autoimmune diseases was not increased following mRNA-based vaccinations, though the frequency of some of them increased after the booster [100]. On the other hand, a growing number of reports point to a possible increased risk of new-onset or worsening of pre-existing autoimmune diseases following vaccination against COVID-19 [100,101,102,103,104]. Case series of autoimmune consequences following COVID-19 vaccination associated with cardiovascular [105,106], hematological [107], hepatic [108], renal [109], neurological [110], and endocrine [111] effects, among others, have been reported. Albeit not yet validated by analytic studies, these signals clearly deserve active monitoring. Consistently, a prospective follow-up study in health-care workers revealed that the plasma level of several autoantibodies increased in strict correlation to the number of vaccine injections [112]. The likely culprit for such adverse reactions is the vaccine spike protein, which can simulate the pathogenic action of the viral counterpart, thereby triggering dysregulated immune responses, probably through molecular mimicry or anti-idiotype mechanisms [72,87,92,113,114,115,116]. As a matter of fact, both the viral and vaccine spike proteins share considerable homology with human proteins or peptides, with an obvious potential to induce autoimmune diseases [117,118,119,120]. It is important to consider these factors when evaluating the potential pathogenic action of vaccines as they may act as contributing causes.

The question of the time window is also critical for the evaluation of the effectiveness and safety of vaccines [121,122]. In general, full vaccine protection (coincident with the highest concentration of anti-Spike IgG) is assumed to start >14 days after the second dose or the booster [123]. Based on this, it has been assumed that prior to this period, the vaccinated individual would be considered as “unvaccinated”, i.e., unprotected and therefore susceptible to infection and infection-related serious consequences, including death. Indeed, in many epidemiologic studies, these individuals are excluded from counting of COVID-19 severe outcomes, including death [124,125]. According to such criterion, also adopted by some governmental health agencies, subjects who died from COVID-19 in the first two weeks after vaccination were considered in the group of the “unvaccinated”.

Along this same line, a recent study has shown that after adjusting for all potential confounding factors, the risk of developing a retinal vascular occlusion significantly increased two years after vaccination with the anti-COVID-19 mRNA pro-vaccine [126].

Thus, an arbitrary time window clearly introduces a distortion in the overall assessment of vaccine efficacy and safety should that vaccine increase the likelihood of serious AEFI.

### 5.4. Did a Specific Test Demonstrate the Causal Role of the Vaccine?

Establishing a laboratory test that can specifically demonstrate the causal link between a new vaccine and an AEFI requires significant time, and this kind of test was not available at the time of anti-COVID-19 vaccination campaign. Currently, two years after the start of vaccinations, it is possible to distinguish the spike protein of the vaccine from that of the virus by mass spectrometry [127]. Thus, when applying the WHO algorithm to a newly developed vaccine, a negative answer to this question should not be considered as evidence of a lack of correlation unless a specific test is available. Another important test that should be considered for a causal role of the vaccine is the serum concentration of antibodies against S- and N-viral antigens. This is important for cases where the AEFI symptoms could be similar to COVID-19 (or post-COVID-19 syndrome) symptoms. In cases where only anti-S antibodies are detected in serum, there is a greater likelihood that the symptoms are actually due to a reaction to the vaccine, as in a recently described case [128]. Similarly, the immunostaining of a panel of anti-viral antigens along with the vaccine-derived S in plasma [72,127] or in the diseased organs (as in autopsies, for instance) may help discriminate the causal link of the AEFI with either the vaccine or the virus [129,130,131,132,133].

Here, it is worth mentioning that autopsy has been shown effective in determining the causal link between COVID-19 vaccination (with ChAdOx1 nCoV-19 by Astrazeneca) and the fatal events that occurred in two individuals 16 and 24 days post-vaccination, respectively [65].

## 6. Scientific Literature and Other Qualifying Factors

**Figure 3:**
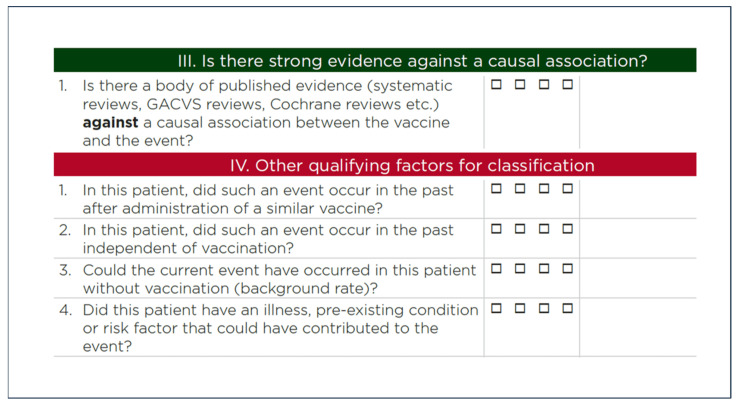
Some questions from the third and fourth steps of the OMS protocol for causality assessment [17]. This work is available under the Creative Commons Attribution-Non Commercial-ShareAlike 3.0 IGO license.

Step III and IV in the algorithm and checklist pose additional questions that require clarification when applied to new generation vaccines (refer to Figure 3).

### 6.1. Evidence Against a Causal Association

The WHO algorithm assesses whether there is published scientific evidence contradicting the causal association between the vaccine and a particular event. Whether this criterion can be applied to COVID-19 vaccines that are made with a new technology is disputable.

In fact, excluding a causal relationship is challenging and, especially for rare events, this can only be achieved after numerous epidemiological studies are included in systematic reviews.

It might be the case that the disease caused by the vaccine emerges clinically after long time, for instance in the case of tumors. Pfizer’s biodistribution study [134] reports, on page 29, a certificate of analysis alongside the *hazard information*, which reads: “WARNING: This product contains a chemical known to the State of California to cause cancer”. This would require at least some years of close monitoring for such potential serious adverse events in human recipients. Compared to solid tumors, hematological tumors can develop faster and can be more easily clinically detected and confirmed with laboratory tests. Some case reports of lymphopoietic tumors [68,135] and a few also for solid tumors [136] temporally associated with COVID-19 mRNA vaccines were reported in the literature as early as 2021.

Thus, to exclude any causal relationship of tumor development with such vaccines, we need more data and more time.

In summary, based on current knowledge, step III cannot be used for those serious AEFIs that are little known in the literature or for which the analytical studies have not yet provided certain answers. Obviously, this limitation applies especially to the early stages after the vaccination rollout and to the most unexpected and rare adverse events.

### 6.2. Did Such an Event Occur in the Past After Administration of a Similar Vaccine?

It is obvious that this question is not applicable to a single patient for new vaccines, especially if made with a novel genetic-based technology, and for diseases not reported for previous “similar” vaccines. It cannot be applied even to previous doses of the same vaccine, because when a serious adverse event occurs and leaves the patient with serious consequences, a subsequent dose of the same product should (hopefully) be avoided.

### 6.3. Did Such an Event Occur in the Past Independent of Vaccination?

This is a logical question, particularly when referring to diseases frequently occurring in the population. As an example, in its manual [17], the WHO discusses skin allergies, common in childhood, and suggests that if dermatitis develops after MMR vaccination, this might well be a coincidence in the absence of a strong and straightforward proof of causality. However, this statement should not be taken as evidence that the vaccine is completely innocent, but rather as a point that the coincidental correlation does not allow one to blame the vaccine without other positive criteria.

### 6.4. Could the Current Event Have Occurred in This Patient Without Vaccination (Background Rate)?

This question also makes sense, apparently. However, one should consider the occurrence of such an event not in absolute terms, but rather in terms of relative frequency. In fact, some diseases, for instance cerebral venous thrombosis and clinical myocarditis, are rare in the general population, but may occur more frequently in vaccinated individuals. This could be related to predisposing specific genetic background [89] or concomitant pathophysiological conditions.

Additionally, there is the case for diseases reported after vaccination that might be relatively frequent even in the unvaccinated population. These include thrombotic, cardiovascular, and autoimmune diseases and tumors. If only 1% of deaths related to these diseases were caused by the vaccine (which would already be a significant increase in absolute terms), this increase might be unnoticed. Therefore, statistical probability cannot be the only criterion used, and the previously mentioned criteria must be considered altogether.

Once again, we must outline that the appearance of a disease in a vaccinated person does not necessarily prove that it has been caused by the vaccine, especially if that disease is relatively common in the general population. However, if the plausibility criterion and time window are respected, this does not disprove the vaccine as the cause.

In conclusion, the usefulness of question IV (Figure 3) as a criterion for assessing causality is questionable, and its uncritical application may lead to misinterpretation of the data and eventually to misinformation.

## 7. Discussion

During a pandemic, mass vaccination is seen as one of the utmost sanitary interventions to protect the population, which implies that patients with any type of disease and healthy people at any age (and with any underlying physiological condition and genetic background) are equally subjected to similar inoculation. Nowadays, the scientific community debates about the need to move from mass toward personalized vaccination, where the subject’s genetic and clinical conditions are taken into consideration for balancing the harm/benefit ratio [89,137,138].

The COVID-19 vaccination campaign in Western countries used genetic-based vaccines, a technology never tested before in humans in such a large scale. When deploying a new vaccine to a large population, safety of the vaccinee is the most important aspect to be considered, even more than efficacy. More types and number of AEFIs are expected to increase with time and the number of vaccinated people. To monitor the safety signals, a variety of institutional databases have been implemented (e.g., the VAERS, V-safe, Vaccine Safety Datalink in US and their analogous versions in other countries). In addition, real world data from population studies have been collected since the beginning of vaccine rollout and continue to accumulate. Safety assessment methods essentially consider dose- and temporal correlations between the vaccine administration and onset of the AEFI, along with consistency in the VAERS, scientific literature, and biological plausibility. It is also important to consider alternative causes that could be responsible for the event.

The analysis and causation of AEFI is a fundamental procedure in pharmacovigilance and has significant implications for vaccination campaigns for maintaining public trust in public health policy. WHO has developed an algorithmic system that consists of logical steps, yet it leaves several questions unanswered. The COVID-19 pandemic has highlighted that current algorithms for assessing causality in AEFI may not be applicable for newly introduced vaccines, nor effective when dealing with rare and unprecedently reported diseases in the short time of its use because of the inapplicability or risk of misinterpretation of some steps.

The WHO manual [17] states in the introduction that “allegations of vaccines/vaccination causing adverse events must be dealt with promptly and effectively. Failure to clearly communicate the risks and benefits of vaccines can undermine public confidence and ultimately lead to lower immunization rates and increased disease incidence”. This intention is appreciated and shared, but it seems to be influenced by the concern that an overestimation of the phenomenon of adverse reactions may also harm vaccination campaigns. If this concern was to influence the choices made in every case of adverse reactions, we might unconsciously attribute most of the pathological phenomena that emerge from pharmacovigilance reports to other causes. This would defeat the dual purpose of pharmacovigilance: to recognize the harm suffered by each individual and to identify specific signals for public health purposes. As we have already mentioned, the WHO manual acknowledges that “Sometimes there are multiple factors that may precipitate the effect (event) or may function as co-factors so that the effect (event) occurs.” This implies that the vaccine itself could be one of the triggering factors leading to the fatal event.

However, in the first step, the algorithm excludes any association if there is another possible cause for the considered event. Part of the responsibility for misunderstandings in the causal analysis in this first step of the algorithm depends on the change in meaning of some words. In fact, the first question of the algorithm is: “*Is there a strong evidence for other causes*”? The evidence must be “strong”, that is, it must provide a trustable proof that the existing disease was the actual cause of death. The whole “question” requires clinical documentation that must “confirm” that this is precisely the cause of death.

This issue was also highlighted by Butt et al., who assessed the likelihood of association between vaccination against SARS-CoV-2 and deaths occurring within 30 days [139]. The authors adopted a modified WHO algorithm, in which the first question asked was whether there was “*clear, alternate, unrelated cause of death identified and documented by a physician*”. We propose that future editions of the WHO text be much clearer on the first step of the algorithm, including the concept that the purported “other cause” should be clearly “unrelated” with any possible pathogenic action of the vaccine. This concept aligns with the plausibility of the mechanism of action, and it was previously suggested by us [32,56] and others [57] for conventional vaccines.

The inefficient nature of passive pharmacovigilance and improper use of the WHO algorithm might have resulted in two deleterious outcomes. At the individual level, many cases of serious fatal adverse effects in which vaccines could have acted as a trigger or co-cause have not been recognized or considered. Dismissing the cause of vaccines in the vast majority of serious adverse events occurring in people with previous illnesses might introduce significant errors in the assessment of the risk/benefit ratio of vaccines, precisely in those categories characterized by greater fragility [1,140,141], as has been shown in a recent post-licensure study where the risk for all-causes of mortality (also accounting for underlying illnesses) has been assessed in a population of vaccinated and unvaccinated individuals living in an Italian province [54].

It is important to note that the conclusions of the WHO algorithm include only three response categories, with no gradations between them. This can lead to misinterpretation, especially since the ‘indeterminate’ category is often confused with ‘not consistent’. When discussing public health impact, it is important to provide a quantitative answer or a range of probabilities on the likelihood that vaccination is the cause. Unfortunately, the WHO algorithm does not allow for a precise level of probability of causal association. Many cases analyzed where certainty cannot be achieved end up in the “Indeterminate” category, which in turn cannot be used for statistical purposes to establish the probability that a certain disease may be associated with vaccinations at the population level.

While this conclusion may be logical in the case of a single event, it has no validity from a public health perspective. Indeed, based on the AIFA Italian database, conclusions about vaccine safety were drawn and disseminated to the public claiming that only 3.6 percent of deaths were attributable to vaccination, while in most cases they were not or were “indeterminate” [66]. Yet, the concept of “indeterminate” is not proof of safety. From a public health point of view, it would be much more useful to be able to calculate the probability that certain events would be attributable to vaccination, to recalculate the risk/benefit ratio taking statistical uncertainty into account, rather than determining the safety based only on a few confirmed cases.

It is becoming evident that COVID-19 vaccinations can also be followed by a subacute or chronic pathological condition, the so-called long post-COVID-19 vaccination syndrome (LPCVS) or post-acute COVID-19 vaccination syndrome (PACVS), which includes general fatigue, muscle and joint pain, numbness of the extremities, orthostatic tachycardia, hypertension, dyspnea, insomnia, anxiety, dizziness, and/or neurological and neuropsychiatric disorders [142,143,144], probably related to autoimmune mechanisms [72,96,97,98,145]. The fact that these effects arise on the basis of genetic predisposition or intrinsic “susceptibilities” should be taken not as an indication against the causal link, but as an indication of the need to understand the existence of possible interactions between the causes. This diagnostic investigation would favor the identification of risk factors and improve pre-vaccination counselling.

Looking ahead, it is necessary to adapt the WHO criteria to greater adherence to new types of infections and of vaccines or to adopt other criteria for causality and the benefit/risk assessments, such as those proposed by Uppsala Monitoring Centre [61] or by the Brighton collaboration [146,147]. To investigate the scientific relationship between COVID-19 vaccines and suspected adverse events, the Korean COVID-19 Vaccine Safety Research Center (CoVaSC) used criteria based on both epidemiological evidence and scientific plausibility [29]. Epidemiological evidence from population studies is classified into four levels, high, moderate, limited, or insufficient, while mechanistic evidence, mainly from biological and clinical studies in animals and humans, is classified as strong, intermediate, weak, or lacking. The method then uses these two types of evidence to draw a conclusion about the causal relationship, which can be described as “convincingly supports” (“evidence established”), “favors acceptance,” “favors rejection,” or “inadequate to accept or reject.” Others have also proposed different methods for computing signals from pharmacovigilance data [148]. In connection with this, it should also be noted that the main methods of evaluating correlation in the field of pharmacology, the WHO-UMC ones and the Naranjo algorithm, include possible conclusions for which the correlation is defined in at least six bands: “Certain”, “Probable/likely”, “Possible”, “Unlikely”, “Unclassified”, or “Unclassifiable” [60].

Other authors, studying the case of Guillain-Barré syndrome, a serious adverse reaction of COVID-19 vaccines [149], have proposed adapting the causality criteria and classifying the extent of the association with the following five categories: “definitely related,” “probably related,” “possibly related,” “unlikely related,” and “definitely not related.” Also, the mentioned study on the deaths after COVID-19 vaccination in Qatar [139] classified the relationship with vaccination as “high probability”, “intermediate probability”, “low probability or not related”, and “indeterminate”. This kind of score has allowed for the drastic reduction of the number of “indeterminate” conclusions, which in the study cited were only 3 out of a total of 138 evaluable ones.

## 8. Conclusions

In conclusion, the application of WHO algorithm for assessing the causal relationship between a COVID-19 genetic vaccine in the first 12–24 months appears inadequate because of the many unknown and unpredictable factors at that time, such as the site, amount and duration of the immunogen synthesis, the pathogenic mechanisms associated with the immunogen, the scarce literature on the subject, the flawed pharmacovigilance for collecting AEFI data, and the short observation period. Though with time such limitations may be partially or totally overcome, the present analysis highlights the need to consider these issues in relation to possible future pandemic events. From that perspective, the method should be revised and made more flexible and adequate to the epidemiological reality and the complex mechanisms of action of the newly introduced immunogenic vaccines. For the purposes of epidemiological research, and therefore for recommendations to the population, the criteria that include a scale of greater or lesser probability of correlation are much more useful than the “indeterminate” conclusion predicted by the WHO algorithm for vaccine products. In future editions of the WHO manual, a greater gradualness of final classifications could allow an improved use of pharmacovigilance data to quantify the risks of new vaccines at a population level.

## Data Availability

Data supporting reported results can be found in cited literature and links to publicly archived datasets.
